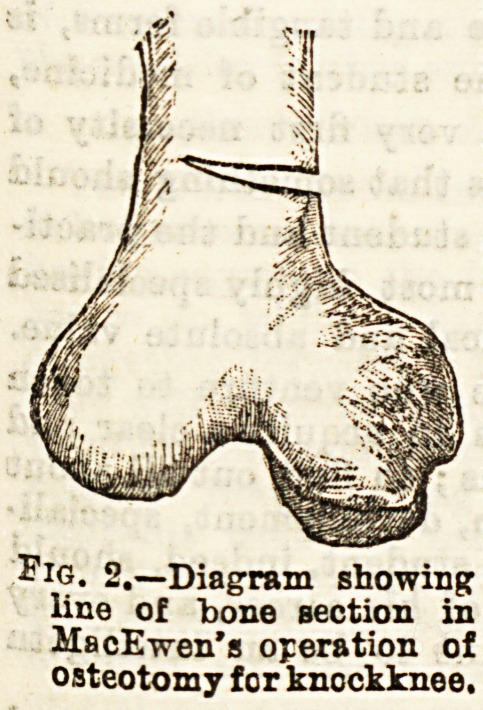# On the Treatment of Knock Knee

**Published:** 1893-01-21

**Authors:** 


### ROYAL INFIRMARY, EDINBURGH.

### On the Treatment of Knock Knee.

The treatment of congenital or acquired deformities —æsthetic surgery—is a subject of great importance to the practical surgeon. Of the conditions demanding such treatment genu valgum or knock knee, on account of its comparative frequency of occurrence, the extent to which it impairs the usefulness of the sufferer, and the ease with which it may be dealt with in its early stages, merits careful consideration.

As the rational treatment of any affection depends on a thorough appreciation of the causes which give rise to it, a brief glance at the etiology of the condition under consideration will not be out of place.

The cases of knock knee which claim the attention of the general practitioner divide themselves into two great classes, one of which may be treated without operation, while the other demands surgical interference.

In the former the sufferers are young children between the ages of two and five, usually the offspring of parents badly developed, and not unfrequently themselves the subject of similar deformity. The children exhibit the general characteristic evidences of rickets, of which, indeed, the knock knee is only a symptom. The weight of the body is out of proportion to the strength of the limbs, and the early attempts at standing or walking cause the weakly bones and ligaments to yield. The misdirection thus produced gradually becomes exaggerated, with the result that the well-known deformity is established. The treatment of such a condition is obvious. In the younger members of this class it is very common to find that suckling has been prolonged far beyond the usual term. A more natural and nutritious diet must be substituted. It should embrace milk, eggs, and raw meat juice. The last named is conveniently made by taking a quarter of a pound of lean raw beef, mincing it very fine, and mixing it thoroughly in a saucer with two tablespoonfuls of cold water. It is left for half-an hour in a cool place, and then strained through a muslin cloth by twisting it. The juice thus obtained is highly nutritious, and not unpleasant to the palate.

The diet of a rickety patient is incomplete which omits cod liver oil. This must be given regularly for some months. It is advantageously combined with some preparation of phosphorous. A teaspoonful of the following mixture given thrice daily shortly after food is found most useful: R. Ol. morrhuæ; syr. calcis lacto-phos.; aq. calcis, āā ℥v.; sodii hypophosphitis, gr. xvj.; mucilaginis, ʒj., ol. cassiæ, mij.; misce. Plenty of fresh air and sunlight are essential, and salt water baths are advantageous.

If the case be seen early, and the deformity be trivial it may be left alone, and will in all probability remedy itself as the child improves under the new régime. In the majority of cases, however, splints must be used to correct the displacement. Complicated and expensive apparatus is quite unnecessary. A long, strong, narrow piece of wood, extending from the axilla to the level of the sole of the foot is evenly padded with wool and covered with cotton. The leg is swathed in boracic lint, all the bony prominences protected with pads or rings of wool, and the splint fixed firmly to the outer side of the limb by strong adhesive plaster. A domette bandage over this maintains and augments the pressure, and, carried round the trunk, fixes the upper end of the splint. The appliance is renewed every two or three weeks. The cases in which this treatment, persistently and conscientiously carried out, fails, are very few, and must be treated on the same principles as those of the second class.

In the other class of valgous patients the deformity is due to quite a different cause. It makes its appearance at the time of adolescence, in youths of a heavy build, who are loosely put together, and who too frequently want that amount of nourishment and support necessary for healthy growth and development. In addition to suffering privation, these patients generally follow some employment which entails prolonged standing or walking, and the carrying of heavy weights, for example, errand boys. The primary lesion is often a straining of the ligaments of the foot, resulting in flat foot, and secondarily to this, the knock knee is established. Once started it tends to increase, and unless measures be taken to arrest it early, a permanent deformity results. Here again, the treatment must be to remove the cause, but herein lies the difficulty, that in removing the cause of his trouble we deprive the patient of the means of earning his bread. A means must be sought of obtaining a more salutary employment, one in which standing and walking are not demanded. An abundance of good, wholesome food must be given, and the general hygienic condition of the patient attended to. Where flat foot exists it must be treated by appropriate means, such as massage, gymnastics for the muscles of the legs, and, if necessary, an artificial support to the instep. The malposition of the knee is to be corrected by a firm external splint on the same principle as that employed in the rickety variety of knock knee.

When the deformity has existed for some years, and the bones begin to solidify, it is out of the question to hope for recovery by such means as have just been mentioned. Nothing short of operative treatment promises a satisfactory result. Fortunately, the certainty with which this operation can be carried out aseptically renders it a comparatively safe one; and the modern operation being exceedingly simple, the patient should seldom be denied the benefit of the surgeon's art.

The operation—osteotomy as it is called—consists in almost completely dividing the shaft of the femur with a chisel, fracturing the remaining portion, and then treating the condition as one of compound fracture, which it practically amounts to.

To describe the operation more in detail. The patient is prepared for the anæsthetic in the usual way. The night before, the legs are thoroughly washed with soap and water, and the region of the knee covered with a carbolised towel. Just before the operation the part is scrubbed with a nail-brush and strong antiseptic. As asepsis is the key to success in this operation too great care cannot be taken in purifying the hands, instruments, dressings, &c. The instruments necessary are an ordinary bistoury, two or three osteotomes (Fig. 1) or chisels of different sizes, a mallet, and a small firm sand-bag enclosed in a carbolised towel

Fig. 1.—OSTEOTOME.

The limb is flexed, and laid on its outer side firmly supported by the sand-bag. A longitudinal incision, about one inch in length, slightly in front of and above the adductor tubercle on the inner aspect of the internal condyle, is made right down to the bone. No normal vessel of importance is cut. The osteotome is passed into the wound, turned so as to lie at right angles to the long axis of the femur, and then by short sharp strokes of the mallet is driven through four-fifths of the thickness of the bone, care being taken to avoid the posterior aspect lest the popliteal vessels be injured. A carbolised swab of wool is placed over the wound, and under cover of it the chisel is withdrawn. The surgeon now forcibly breaks through the rest of the thickness of the femur, and places the limb in a good position. It is found advisable to over-correct the displacement, as otherwise during the process of repair a slight degree of deformity is sometimes reproduced.

FIG. 2.—Diagram showing line of bone section in MacEwen's operation of osteotomy for knockknee.

Two or three catgut stitches are used to close the wound in the soft tissues and facilitate primary union, a dry antiseptic dressing is applied, and the limb immobilised by being encased in plaster of Paris from the toes up to the groin, a small window being left to facilitate the examination and, if necessary, the dressing of the wound. The original plaster case is replaced at the end of four or six weeks by a lighter appliance, such as starch or poroplastic, and the patient goes about on crutches till the bones are sufficiently consolidated to sustain his weight.

## Figures and Tables

**Fig. 1. f1:**
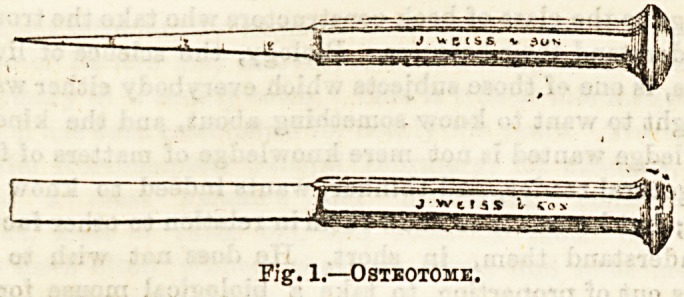


**Fig. 2. f2:**